# Modulated critical currents of spin-transfer torque-induced resistance changes in NiCu/Cu multilayered nanowires

**DOI:** 10.3762/bjnano.15.32

**Published:** 2024-04-03

**Authors:** Mengqi Fu, Roman Hartmann, Julian Braun, Sergej Andreev, Torsten Pietsch, Elke Scheer

**Affiliations:** 1 Fachbereich Physik, Universität Konstanz, 78457 Konstanz, Germany

**Keywords:** AAO template, critical current, multilayered magnetic nanowires, spin-transfer torque, three-dimensional devices

## Abstract

We present a novel method combining anodic aluminum oxide template synthesis and nanolithography to selectively deposit vertically patterned magnetic nanowires on a Si substrate. With this approach we fabricated three-dimensional nanowire-based spin valve devices without the need of complex etching processes or additional spacer coating. Through this method, we successfully obtained NiCu/Cu multilayered nanowire arrays with a controlled sequence along the long axis of the nanowires. Both magnetic switching and excitation phenomena driven by spin-polarized currents were clearly demonstrated in our NiCu/Cu multilayered nanowires. Moreover, the critical currents for switching and excitation were observed to be modulated in an oscillatory manner by the magnetic field in the nanowire-based devices. We present a toy model to qualitatively explain these observations.

## Introduction

Spin-transfer torque (STT) has been utilized as an effective tool in fundamental studies [1–2] as well as advanced technologies [2–6] to quickly change the magnetization in nanoscopic magnetic systems [2,5,7]. Among manifold material synthesis strategies, high-aspect-ratio multilayered nanowire arrays based on anodic aluminum oxide (AAO) template-assisted electrodeposition has attracted wide interest because of its low cost as well as high flexibility on tailoring the magnetic properties of magnetic systems and thus STT effects [8–12]. Moreover, it enables a larger number of free layers, whose magnetization can be flipped by STT [10] in one nanowire [9,11]. Comparing with the classical trilayer system (including one hard layer with fixed magnetization, one free layer, and one nonmagnetic spacer layer in between) [10], the presence of more free layers enhances, for example, the magnetoresistance (MR) [7,13] and ferromagnetic resonance modes [14–15]. Meanwhile it has been shown that it might also lead to sequential flipping of different free layers, which, on the one hand, increases the complexity of the STT effects [16] and, on the other hand, expands its applications [5].

In this work, we present a nonmonotonic dependence between the critical current of STT-assisted resistance changes and the strength of the external magnetic field in NiCu/Cu multilayered nanowire devices with arbitrary sequence of magnetic and nonmagnetic sections along the long axis of the nanowires. The STT devices were fabricated through a newly developed method, which enables to selectively deposit the magnetic nanowires on the Si substrate and to fabricate three-dimensional (3D) devices contacting a few or even single nanowires without complex etching processes [17] or additional spacer coating [18–19].

## Results and Discussion

**Figure 1:**
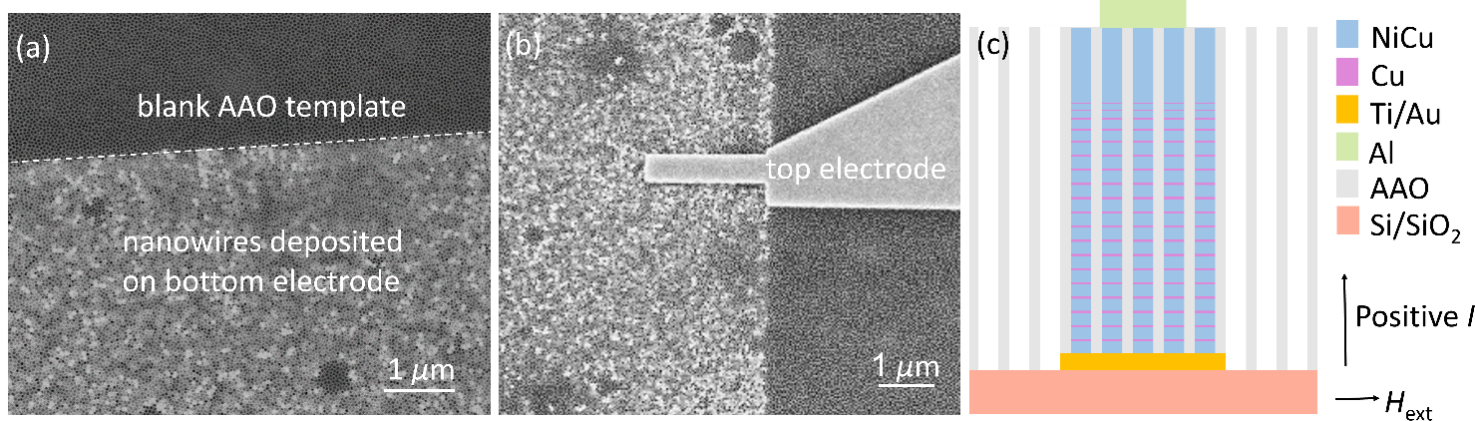
(a) SEM image after nanowire deposition and surface polishing. The bright dots represent the deposited nanowires the tops of which are near the surface of the AAO template. (b) SEM image of nanowire-based devices. The measured nanowire array was contacted by the patterned Au bottom electrode and the Al top electrode. (c) Sketch of the cross section of the device.

The AAO template was fabricated by directly anodizing a ca. 1 µm thick aluminum (Al) film on a silicon (Si) substrate covered with 200 nm SiO_2_ and patterned Ti/Au (5/50 nm) bottom electrodes. It has pores with a diameter of around 35 nm, an interpore distance of around 50 nm, and a height of around 1 µm. The electrodeposition of multilayered nanowires was carried out in situ using a three-electrode potentiostat in the pulsed mode [20] at 25 °C. Note that the nanowires were selectively deposited in the pores on the top of the Ti/Au bottom electrodes as shown in Figure 1a. Therefore, most of the surface area of the AAO template (or Si substrate) is isolated from the bottom electrodes and the magnetic nanowires, thereby largely improving the flexibility for the design of the top electrodes. After removing the overgrowth by a thin blade or milling in argon plasma, a thick Al film of 180 nm was patterned to build the top electrode by thermal evaporation at a large deposition rate (>3 Å/s) to ensure quick and continuous film formation and, thus, to efficiently avoid Al to be deposited into the pores. Therefore, only the nanowires the top of which have reached the upper surface of the AAO template were contacted with the top electrodes. After a lift-off process, the 3D devices based on the NiCu/Cu multilayered nanowire array were obtained. A typical device structure is shown in Figure 1b,c. The description of the setup and more details of AAO template fabrication, electrodeposition and device fabrication, and scanning electron microscopy (SEM) images of the devices during the fabrication process are presented in Supporting Information File 1.

In most reported works, the nanowires were deposited in all pores of the AAO templates [18–21]. Additional etching steps or coating steps were necessary to define the contact area and to obtain a small contact array, which complicates the fabrication process [18–19]. Compared with these works, our process does not need any additional etching step to either define the contact area of the nanowire or electrodes for the top contact. Therefore, the fabrication process of 3D devices becomes easier.

**Figure 2:**
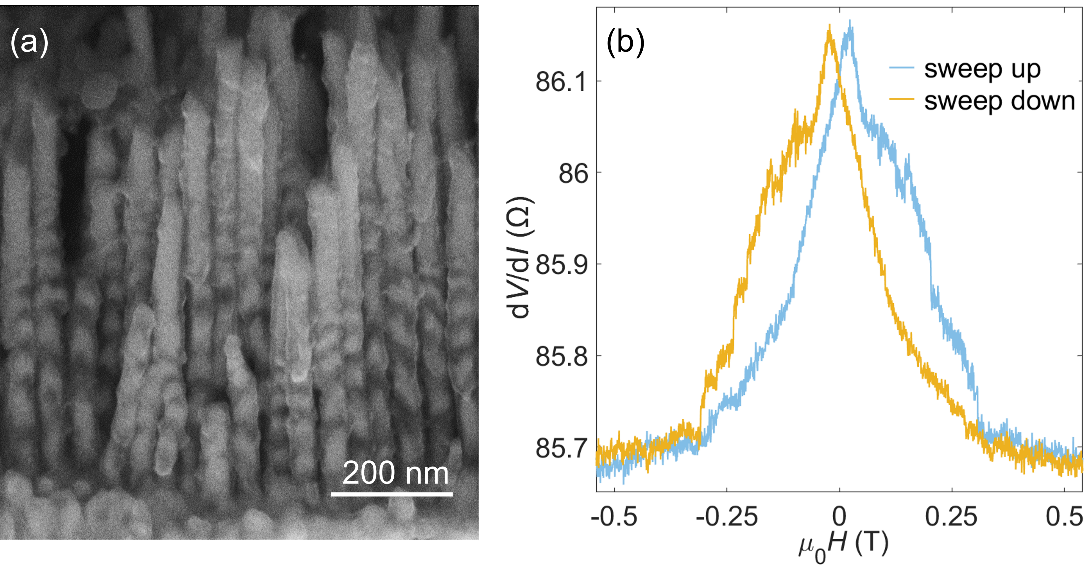
(a) SEM image of nanowires after the AAO template was removed. The Si substrate was tilted by 60° when taking the image. (b) Hysteretic behavior of the d*V*/d*I* curve of the nanowires upon sweeping of the magnetic field.

Figure 2a shows a SEM image of nanowires after removing the AAO template and top electrodes by diluted NaOH solution. Each nanowire consists of multiple NiCu layers of different thickness that are separated by thin Cu layers (denoted as Cu spacer in the following). From the bottom to the top, both NiCu layers and Cu spacers get thinner, which can be caused by the dynamical change of the ion concentration in the holes. Eventually, the thickness of the Cu spacers becomes zero and no well-defined Cu spacer can be observed. Through the SEM characterization, the thickness of the bottom NiCu layers is estimated to be around 22 nm and is reduced to below 10 nm towards the upper end of the nanowire. Only NiCu is deposited in the pores near the surface of the AAO template forming a long segment of NiCu on the very top of the nanowires. This varying structure of the magnetic and spacer layers along the long axis of the nanowires leads to variations of the magnetization in different magnetic layers and to spin accumulation in the spacer layers. Consequently, we expect distinct magnetic and magneto-electrical features compared to the reported nanowires with evenly spaced magnetic layers.

The magnetic and magneto-electrical properties of the multilayered nanowire array were characterized in a conventional bath cryostat at a temperature of 4.2 K. The differential resistance (d*V/*d*I*) was measured using the lock-in technique with a modulation voltage (5 mV) added to a DC bias voltage at a frequency of 4531 Hz. A four-point measurement was used to exclude the effects of the cabling. A positive current is defined such that the electrons flow from the top electrode to the bottom electrode. A magnetic field was applied perpendicular to the nanowires (Figure 1c), which is the easy axis according to the disk-like shape of the NiCu layers and the vibrating sample magnetometer (VSM) measurements (see Supporting Information File 1). In all measurements, the magnetic field amplitude was first set to a large initial value (usually 2 T or −2 T, depending on the direction of the sweep) to align the magnetization of all magnetic layers before sweeping the magnetic field.

Figure 2b shows a differential MR curve of a device that presents hysteretic behavior and a total MR change of about 0.45 Ω (ca. 0.52% of the total resistance) over the field sweep. The number of contacted nanowires can be estimated to be around six in the present case; for detailed calculations see Supporting Information File 1. The maximal d*V*/d*I* is achieved at µ_0_*H* ≈ −21 mT/21 mT in the downsweep/upsweep of the magnetic field, respectively. The gradual changes in the d*V/*d*I* hysteresis loop may be attributed to multidomain structures in the long NiCu segment and wire-to-wire variations of the interaction between the segments of the nanowires. Several jumps and drops are observed, which are mainly mirror-symmetrically distributed in the upsweep and downsweep curves. Since d*V/*d*I* depends on the angles between the magnetization directions of all the neighboring magnetic layers, these jumps and drops indicate that the magnetization of multiple NiCu layers does not uniformly change but rather flips one by one under different magnetic fields. Intermediate configurations of magnetic layers except for the fully antiparallel (AP) and fully parallel (P) configurations can exist; therefore, more than one free layer might contribute to the MR and STT effects discussed below. They can be understood by the unequal coercivities and interaction of different NiCu layers due to the varying thickness of the NiCu and Cu layers [10,21].

To be specific, as the size of the magnetic layer (especially for soft magnets as NiCu) continues to shrink below a critical value, its magnetization is increasingly affected by thermal fluctuations, and its coercivity shrinks [22]. It has been reported that the coercivity as well as the angular momentum of NiCu become smaller when the thickness is reduced to several to tens of nanometers [22–25]. Thus, the magnetic field to reverse the magnetization of the different magnetic layers within one nanowire can vary significantly. In addition, the different thickness of the Cu spacer layers largely influences the interactions, such as the exchange energy between neighboring magnetic layers [24,26], and further increases the sequential changes of the magnetization direction of the different magnetic layers during the magnetic field sweep. Therefore, when the magnetic field is not large enough to align the magnetization of all magnetic layers, the magnetization of the neighboring NiCu layers is partly antiparallel and partly parallel, forming intermediate configurations of the magnetization in the nanowires. Generally, configurations with neighboring magnetic layers being mainly oriented parallel to each other show a lower resistance [16]. Since the STT originates from the imbalance of incident, transmitted, and reflected spin currents at the interfaces of the magnetic system [7], the varying sequence of magnetic and spacer layers in the current direction are also instrumental to induce STT and, thus, to the current-induced change of d*V*/d*I* in the device.

**Figure 3:**
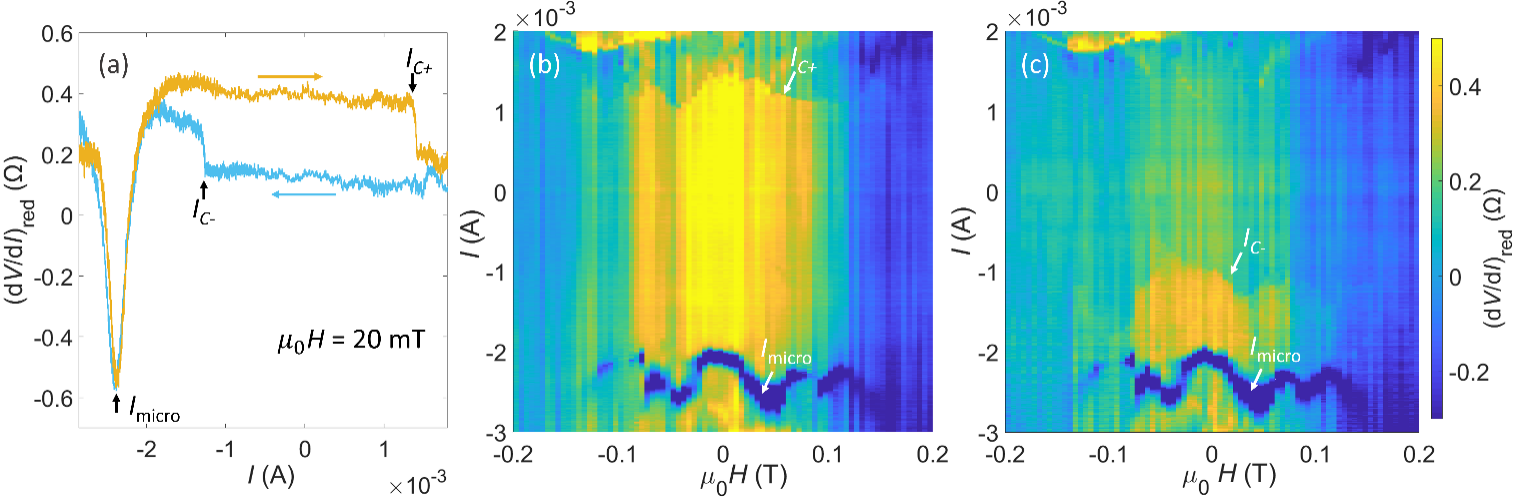
(a) The hysteresis of the reduced differential resistance (d*V*/d*I*)_red_ under current sweep. The magnetic field was first set to 2 T to form the P state of the nanowires and then ramped down to µ_0_*H* = 20 mT. (b, c) 2D color maps of (d*V*/d*I*)_red_ as a function of magnetic field and current. The current was ramped up in (b) and was ramped down in (c).

In the device studied here, a hysteretic d*V*/d*I* as a function of the applied current *I* and a reversible, non-hysteretic dip of the d*V*/d*I* signaling current-induced STT have been observed. Both features are modulated in an oscillatory manner by the applied magnetic field, as shown in Figure 3 for the downsweep of the magnetic field and in Figure S13 (Supporting Information File 1) for the upsweep of the magnetic field. As *I* increases, a continuous increase in d*V*/d*I* was observed in the raw data (Figure S11a, Supporting Information File 1). It results from Joule heating due to the high current density rather than from spin-related effects. Therefore, in order to visualize the spin-related features, the reduced differential resistance (d*V*/d*I*)_red_ was obtained by subtracting the background curve measured at µ_0_*H* = −0.3 T from the raw data. Note that the spin-induced features also can be observed in the raw (d*V*/d*I*)–*I* curves under different µ_0_*H* (see Figure S11b of Supporting Information File 1). Figure 3a shows a typical current dependence of (d*V*/d*I*)_red_ at µ_0_*H* = 20 mT, which shows an abrupt drop of around 0.19 Ω at a positive critical current *I*_c+_ of 1.5 mA in the upsweep of *I* (initiating from a large negative current) and an abrupt jump at a negative critical current *I*_c−_ of around −1.2 mA in the downsweep of *I* (starting from a large positive current), thereby forming a hysteresis loop. The hysteresis loop and the abrupt change of (d*V*/d*I*)_red_ indicate that the free layers remain single domains (so-called “macrospin approximation”) and are flipped uniformly during the switching by the current-induced STT [7,27]. By analyzing the dependence of the (d*V*/d*I*)_red_ change on the direction of current sweep, the topmost NiCu layer can be further verified to be the fixed layer and the thin NiCu layers underneath as free layers [7]. When *I* is swept to more negative values, a reversible large dip of the (d*V*/d*I*)_red_ is observed around −2.3 mA in both current sweep directions. This reversible dip indicates that the macrospin approximation of free layers breaks down to nonuniform excitations, which means the magnetization of the ferromagnet is spatially nonuniform [27–28]. Nonuniform excitations occur in a very thin magnetic layer when the spin accumulation on its two sides is different. Under the critical current (*I*_micro_), the transverse instability makes the magnetization change along the interface, opening low-resistivity paths for both spin-up and spin-down electrons at different spatial positions of the magnetic layer, and thus reduces (d*V*/d*I*)_red_. The current densities of *I*_c+_, *I*_c−_, and *I*_micro_ are estimated to be of the order of 10^7^ A/cm^2^, which is comparable to the reported values in trilayer [18,29] or multilayer [16,26] magnetic nanowires. For more details of the signatures of STT in the hysteresis loops and the estimation method of the current density see Supporting Information File 1.

*I*_c+_, *I*_c−_, and *I*_micro_ are modulated and present an oscillatory behavior with magnetic field as shown in the two-dimensional (2D) color maps of Figure 3b,c. The functional shape of *I*_c+_, *I*_c−_, and *I*_micro_ shows several maxima and minima in both Figure 3b and Figure 3c. The modulated STT features are mainly located in the low-magnetic-field region between −0.15 T and 0.16 T, where (d*V*/d*I*)_red_ changes most strongly with the magnetic field and the magnetizations of the NiCu layers are partly antiparallel. Indicated by several abrupt changes of (d*V*/d*I*)_red_ in the hysteresis curve in Figure 2b, the thinner NiCu layers on the top of the nanowires are flipped one by one under small magnetic fields; thus, more than one free layer can exist in the measured device [8,30–32]. Since *I*_c+_, *I*_c−_, and *I*_micro_ are closely related to the properties (e.g., thickness, volume, coercivity, and angular momentum) of the free layers and the spin accumulation at the interfaces between the different layers [7,26,28], more free layers are expected to bring more complex dependencies of the critical currents on the magnetic field, compared to a trilayer system whose *I*_c+_, *I*_c−_, and *I*_micro_ values are reported to monotonously change with the applied magnetic field [28,33]. To our knowledge, this oscillatory behavior of the critical currents in multilayer nanowire arrays is rarely reported.

**Figure 4:**
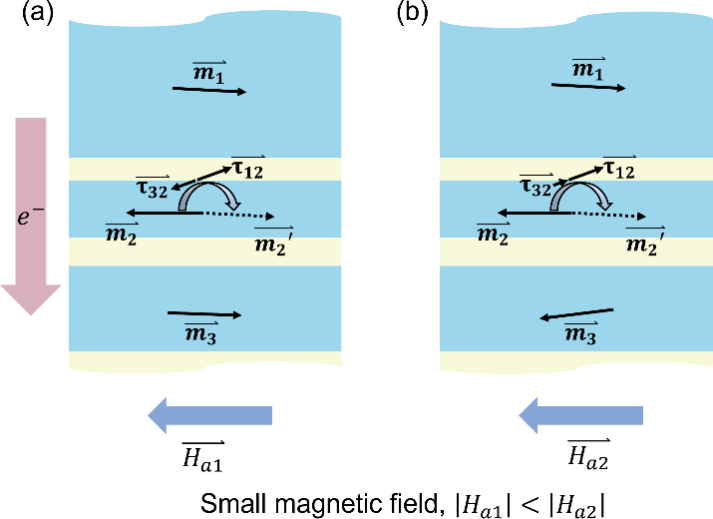
Configurations of the magnetization of NiCu layers and STT applied to the free layers under different magnetic fields.

To phenomenologically interpret the nonmonotonous changes of the critical currents, we propose a model system consisting of five adjacent layers (one fixed layer, two free layers, and two spacer layers) as shown in Figure 4. With this model we qualitatively explain how one period of the *I*_c+_ modulation is formed. As shown in Figure 4, the NiCu layers are labelled as layer 1 to layer 3 from top to bottom. As discussed above, layer 1 is the fixed layer, and layers 2 and 3 are the free layers. The Cu spacer between layers 1 and 2 is labelled as spacer 1 and that between layers 2 and 3 as spacer 2.

When µ_0_*H* is around or slightly above 0 T, as shown in the MR curve (yellow line) in Figure 2b, d*V*/d*I* has a relatively high value, which represents an AP state among layers 1 to 3 (configuration 1 in Figure 4a). When µ_0_*H* is swept from above 0 T to a small negative value *H*_a1_, configuration 1 remains, while the effective magnetic field *H*_eff_ on layer 2 becomes larger. Here, *H*_eff_ is determined by the external magnetic field as well as the exchange stiffness, the dipolar stray field, and the anisotropy field caused by the spin–orbit interaction [34]. Since the magnetization of layer 1 (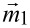) and layer 3 (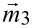) are both parallel to the applied magnetic field (here: positive), the exchange stiffness as well as the dipolar stray field on layer 2 point in the opposite direction (negative magnetic fields) It makes *H*_eff_ on layer 2 negative even when µ_0_*H* is slightly positive; it becomes more negative during the downward sweep of µ_0_*H*. The damping term in the Landau–Lifshitz–Gilbert–Slonczewski equation increases as *H*_eff_ becomes larger. Therefore, as long as configuration 1 remains, *I*_c+_ turns more positive as µ_0_*H* becomes more negative.

When the magnetic field becomes more negative (|*H*_a1_| < |*H*_a2_|) and 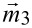 is flipped (labelled as configuration 2 in Figure 4b), the direction of the exchange coupling as well as of the dipolar stray field exerted by layer 3 to layer 2 is inverted, which decreases *H*_eff_ and, thus, *I*_c+_. At the same time, the STT exerted on layer 2 by layer 3 (labelled as 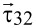 in Figure 4) favors the flip of layer 2. A smaller current is required in configuration 2 than in configuration 1 to flip 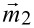, which further decreases *I*_c+_. The continuous change of *I*_c+_ might be caused by the continuous rotation of 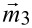 or the dipolar interaction between the nanowires (a more extended discussion can be found in Supporting Information File 1). Similarly, *H*_eff_ increases again when µ_0_*H* is more negative as long as configuration 2 remains. These changes on *H*_eff_ and 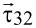 between configuration 1 and 2 lead to one oscillation period of *I*_c+_. A similar argumentation holds for *I*_c−_ and *I*_micro_. Note that for the whole features under µ_0_*H* between −0.15 T and 0.16 T in Figure 3b and Figure 3c, the flipping and nonuniform excitation process would be even more complex, and further studies are needed to describe the phenomena completely.

## Conclusion

In summary, we developed a new method including traditional AAO template fabrication, electrodeposition, and nanolithography-based techniques to selectively deposit NiCu/Cu multilayered nanowire arrays with nonuniform pattern along their axis on a Si substrate. Thus, we fabricated three-dimensional STT devices without complex etching processes or additional coating. The device discussed in detail presents a total magnetoresistance of about 0.45 Ω (ca. 0.52% of the total d*V*/d*I*) at 4 K. In addition, hysteretic behavior and a reversible dip of d*V*/d*I* under current sweeping, indicating a flip of the magnetization and nonuniform excitation assisted by current-induced STT in the thin NiCu layers, have been observed to oscillate when varying the applied magnetic field. Based on a model system with simplified layer structure, we show that the modulation of the STT-induced features can be interpreted as asynchronous changes of the magnetization direction of different NiCu layers as well as spin accumulation at different interfaces.

## Supporting Information

See Supporting Information File 1 for a detailed description of the setups of AAO template preparation and electrolytic nanowire deposition, an estimation of the thickness of NiCu and Cu layers and the current density in the measured device, a detailed description of STT-assisted resistance switching in multilayered nanowires, raw data before the background subtraction, a schematic diagram of the four-point measurements at low temperature, 2D color maps of (d*V*/d*I*)_red_ over a larger current range and under upsweep of the magnetic field, the potential influence of the dipolar interactions among nanowires, VSM measurements for the electrodeposited nanowire arrays in AAO template, and a discussion on the wire-to-wire variation.

File 1 Additional experimental data.

## Data Availability

All data that supports the findings of this study is available in the published article and/or the supporting information to this article.
