# Supplementary material for: Modulated critical currents of spin-transfer torque-induced resistance changes in NiCu/Cu multilayered nanowires
Source: Beilstein J Nanotechnol. 2024 Apr 3;15:360–6. doi: 10.3762/bjnano.15.32 (PMC10999983; doi:10.3762/bjnano.15.32)
Supplement: File 1 — Additional experimental data. [file Beilstein_J_Nanotechnol-15-360-s001.pdf]

BEILSTEIN JOURNAL OF NANOTECHNOLOGY

Supporting Information

for

Modulated critical currents of spin-transfer torque-induced resistance changes in NiCu/Cu multilayered nanowires

Mengqi Fu, Roman Hartmann, Julian Braun, Sergej Andreev, Torsten Pietsch and Elke Scheer

Beilstein J. Nanotechnol. **2024,** *15,* 360–366. doi:10.3762/bjnano.15.32

Additional experimental data

License and Terms: This is a supporting information file under the terms of the Creative Commons Attribution License (https://creativecommons.org/licenses/by/4.0). Please note that the reuse, redistribution and reproduction in particular requires that the author(s) and source are credited and that individual graphics may be subject to special legal provisions.
The license is subject to the *Beilstein Journal of Nanotechnology* terms and conditions: (https://www.beilstein-journals.org/bjnano/terms)

1 Setup of AAO template preparation

The sample fabrication was started with a silicon (Si) chip coated with 200 nm SiO_2 and a side length of 7 mm. The bottom Ti/Au (5/50 nm) electrodes and the markers used to locate the bottom electrode in further fabrication processes were first patterned by electron-beam lithography (EBL) and deposited by electron-beam evaporation (EBE) on the Si chip. After a standard lift-off process, a highly pure aluminum (Al) layer with thickness around 1 μm was deposited by thermal evaporation. The quality of the Al layer is decisive for the subsequent quality of the AAO template. To increase the quality, the Si chip was heated to at least 150 °C before the Al deposition to evaporate the adhesive water and organic residues from the surface of Si chip and the chamber vacuum before deposition was below 10^{-8} mbar.

As shown in Figure S1, the Si chip was then fixed and attached to a copper block by vacuum and a metal spring. The copper block functioned as both heat sink and cathode and was connected to a Peltier element, whose temperature was precisely controlled by a PID controller. Then, a Teflon cell was gently mounted onto the top surface of the Si chip and sealed by an O-ring (labelled as O-ring seal 1) to form a closed container (labelled as Teflon container 1) for the solutions used in the AAO template fabrication. Then, the sulfuric acid electrolyte (0.3 M H_2SO_4, 10% ethanol) was filled into the Teflon container 1, and the anode electrode made of a platinum wire was inserted into the sulfuric acid electrolyte. Then, the Si chip and the sulfuric acid electrolyte were cooled down to 1 °C by the Peltier element, and after their temperature had been stabilized, the anodization was started by applying 20 V potential difference between the anode electrode and the Al layer (with a Yokogawa GS610 Source Measure Unit, also measuring the current at the same time).

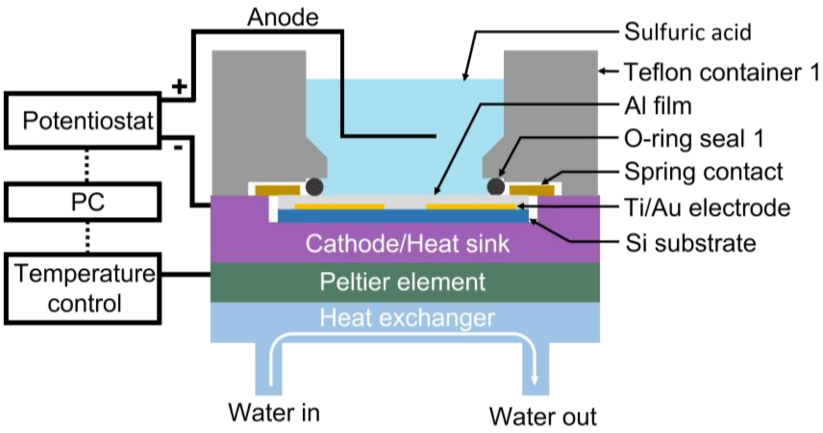

Figure S1: Schematic image of the setup used for AAO template preparation.

To obtain a high-quality AAO template, we employed the two-step anodization method. The first anodization step was used to form a well-ordered concave pattern on the surface of the Al layer, which acted as a self-assembled mask for the second anodization step. An additional function of the first anodization step is to control the total height of the AAO template to range from 1 µm to 200 nm by varying its anodization time. In the electrically measured device shown in the main text, the first anodization lasted 675 s. Note that usually the first anodization step needs to be long enough to obtain the well-ordered concave pattern.

To expose the well-ordered concave pattern to the sulfuric acid electrolyte in the second anodization step, the alumina layer generated in the first anodization step was then removed by filling the etching solution (0.45 M CrO_3 in 3.5 vol % phosphoric acid) into the Teflon container 1 and maintaining the temperature at 50 °C for 20 min. When the alumina layer was completely etched, the etching solution was removed, and new sulfuric acid electrolyte was filled into the Teflon container 1.

The second anodization started by repeating with the same parameters as in the first anodization step, except that the end point of the second anodization can be determined by observing a distinct

color change of the substrate instead of a fixed anodization time. Usually, we stopped the process when the layer above the bottom electrode pattern became transparent and, therefore, the electrode pattern could be clearly observed. At this end point, the anodized template reached the surface of the bottom Au electrodes at the area of the electrode or the SiO_2 layer where it was not covered with the metal film. As soon as the anodized layer reached the bottom Au electrodes, the anodic current started to increase because the alumina barrier between the pores and the Au electrodes became thinner. And after tens of or more than one hundred seconds, the anodic current was relatively stable, which was another criterion for the end point of the second anodization step, as shown in Figure S2b. The thin alumina barrier was then removed by 5% phosphoric acid at 25 °C for 11 min, so that the pores subsequently penetrated to the surface of the Au layer and, at the same time, were widened to a diameter of around 35 nm. The height of the pores is around 1 μm, and the interpore distance is around 50 nm.

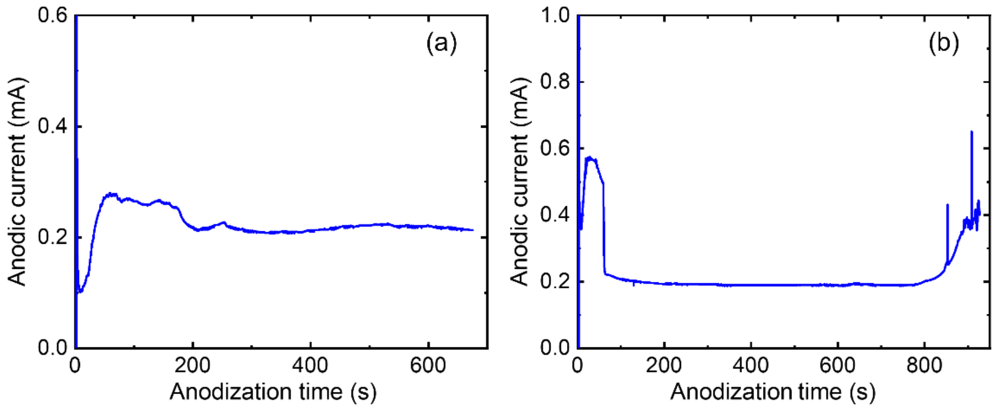

Figure S2: The anodic current versus anodization time in the first (a) and second (b) step of the anodization process of the AAO template.

2 Setup of electrolytic nanowire deposition

After the contact areas have been etched free and the barrier oxide has been removed, metal can be deposited into the pores. The setup used in the nanowire deposition was similar to that for the AAO template fabrication, except that the Teflon container (labelled as Teflon container 2) as well as the O-ring (labelled as O-ring 2) are intentionally designed to be smaller, and a reference electrode was added into the electrolyte, as shown in Figure S3. The deposition of nanowires was carried out using a three-electrode potentiostat in the pulsed mode with a saturated Ag/AgCl electrode as the reference electrode, the bottom Ti/Au electrode under the AAO template as the working electrode, and the platinum wire (forming a ring shape) as the counter electrode. The electrolyte used for the NiCu/Cu multilayer deposition was composed of 0.17 mol/L $NiSO_4 \cdot 6H_2O$, 0.03 mol/L $NiCl_2 \cdot 6H_2O$, 0.12 mol/L H_3BO_3, and 0.02 mol/L $CuSO_4 \cdot 5H_2O$. The pH value of the electrolyte was adjusted to pH 3–4 by adding 0.3 M H_2SO_4. Potentiodynamic studies were conducted to determine the deposition potentials for the NiCu and Cu layers. In this work, the potentials used for NiCu and Cu were −1.05 V for 1 s and −0.3 V for 5 s at 25 °C, respectively, as shown in Figure S4. Pulse cycles were repeated about 75 times and a bulk of overgrowth can be observed on the surface of the AAO template as shown in the insert of Figure S5a. Though we used constant pulse conditions during the whole electrodeposition process, the generated nanowires do not show uniform NiCu/Cu multilayers along the axial direction, especially at the upper part of the nanowires. Similar irregular structures have been widely observed in different batches of nanowire arrays by using deposition parameters similar to those in our experiment. These nonuniform multilayer structures in electrodeposition have been widely reported [1] and are attributed to several reasons. For instance, the ion concentration changes near the surface of

nanowires [2], hydrogen evolution or other parameters differ and, thus, the corrosion effect becomes more severe during the deposition process of nanowires [3].

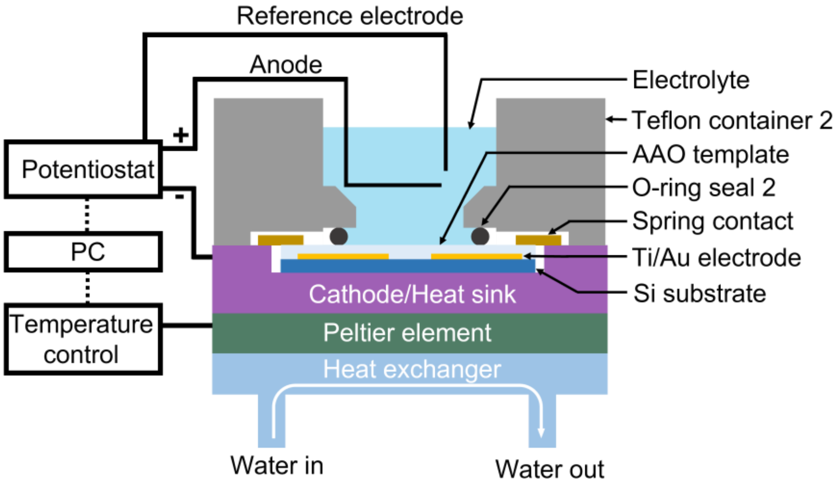

Figure S3: Schematic image of the setup used for nanowire electrolytic deposition.

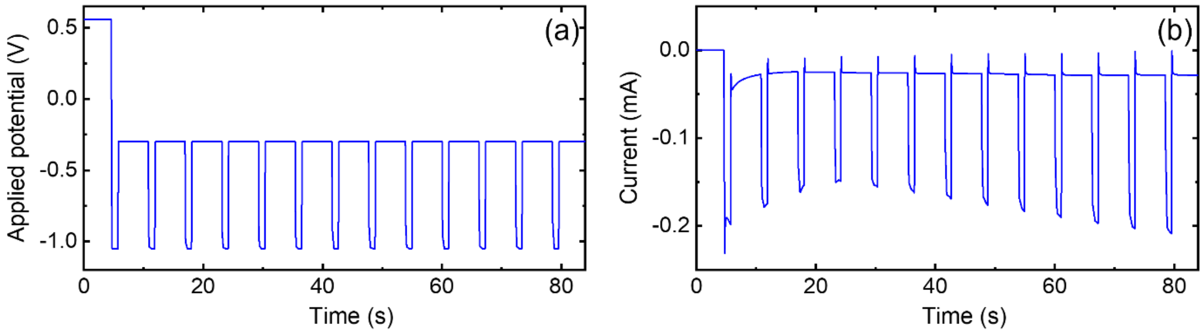

Figure S4: The applied potential (a) and anodization current (b) during the electrodeposition of the NiCu/Cu multilayer nanowire.

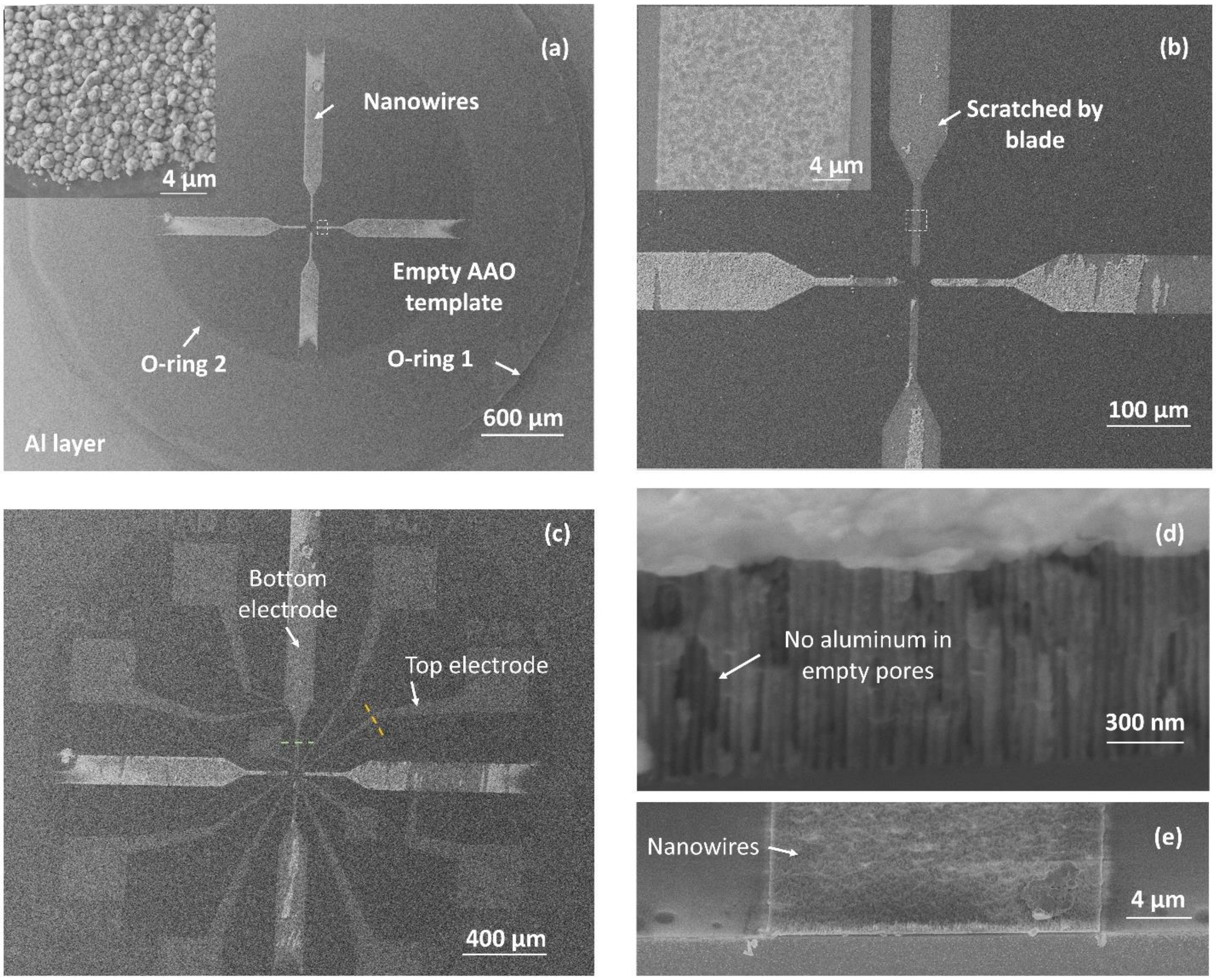

Figure S5: SEM images of structures during the fabrication processes of vertical nanowire-based devices. (a) SEM image of the devices after AAO template fabrication and nanowire deposition. The AAO template was formed inside the area enclosed by the O-ring 1. Outside the O-ring 1 was the thick Al layer, which was electrically connected with the bottom electrode. Because of the lack of the broadening process, the holes of the AAO template between O-ring 1 and O-ring 2 had much smaller diameter than those of the AAO template inside the O-ring 2, which leads to a contrast difference between these two regimes in the SEM image. The nanowire arrays were only deposited into the AAO template above the bottom electrodes and inside the area enclosed by the O-ring 2. The insert figure is a zoom-in image of the area enclosed by the dashed line of (a). An overgrowth of metal bulk can be observed on the surface of the AAO template. (b) SEM image of the devices after removing the overgrowth of metal bulk by softly scratching the surface of AAO template. The insert is the zoom-in image of the area enclosed by the dashed line of (b) and shows that the overgrowth was removed and the tops of the nanowires are exposed. (c) SEM of the devices after the fabrication of the top electrodes. (d) SEM image of the cross section of the yellow dashed line in (c). No Al was deposited into the holes of AAO template. (e) SEM image of the nanowire arrays

after AAO was removed by NaOH solution. The silicon wafer was cracked near the green dashed line of (c), and the nanowire arrays can only be observed at the top of the Ti/Au bottom electrode.

The deposition area can be precisely controlled by the pattern of the bottom Au electrode and the location of the O-ring 2. It is worth noting that the nanowires were only deposited in the pores that were on the top of the bottom Ti/Au electrodes according to the SEM characterization (Figure S5a,e). Both the markers and the SiO_2 surface were free of nanowires since they were not exposed to the potential of the working electrode during the electrodeposition process. Furthermore, during the whole processes of the AAO template fabrication and nanowire deposition, the position of the Si chip was fixed. As shown in Figure S5a, the deposition area of the nanowires was centered with respect to the area of the AAO template. Yet, the area of the AAO template, which was determined by the diameter of O-ring 1, was much larger than that of nanowire deposition. Therefore, most of the surface of the AAO template (or Si substrate) were isolated from the bottom electrodes and the nanowires and left large space for the top contacts, largely improving the flexibility for the design of the top electrodes.

Before the fabrication of the top electrodes, the overgrowth of the AAO template had to be removed to expose the top surface of the metal nanowire either by softly scratching with a thin blade (as shown in Figure S5b) or milling with an argon plasma (as shown in Figure 1a). Then standard EBL and development processes were used to transfer the designed pattern of top electrodes onto the surface of the AAO templates. A thick Al film of 180 nm was deposited as the top electrode by thermal evaporation at large deposition rate (>3 Å/s). Note that the large deposition rate in this step is necessary to ensure quick and continuous film formation of Al; also it efficiently avoided Al deposition into the pores (as shown in Figure S5d). Therefore, only the nanowires whose top have reached the upper surface of the AAO templates were contacted with

the top electrode, and the length of the contacted nanowires in the devices is determined by the height of the AAO template. After a lift-off process the fabrication of the three-dimensional (3D) devices based on NiCu/Cu multilayer nanowire arrays was finished (as shown in Figure S5c).

3 Estimation of the thickness of the NiCu and Cu layers

We can estimate the thickness of the NiCu and Cu layers at the lower end of the nanowires to be around 22 and 7 nm, respectively, based on our SEM observations of several samples with similar growth parameters for the bottom part of the nanowires. As for the top part of the nanowires, according to the SEM image in Figure 2a and Figure S6, we know that the thickness of the NiCu layers is reduced to below 10 nm towards the upper end of the nanowire. However, limited by the resolution of the SEM, we cannot provide a precise estimation of the thickness of the NiCu layers. Based on the abovementioned estimation on the thickness of the NiCu/Cu layer, the number of NiCu/Cu layers of the nanowires studied in Figure 2 and Figure 3 is larger than 28, if we consider the total length of nanowires to be around 1 μm and the long segment in the top of the nanowires to be around 200 nm. Since an overgrowth was observed, and the holes in AAO template were completely filled before the 50 cycles of electrodeposition were finished, the number of Ni/NiCu layers was estimated to be between 28 and 50.

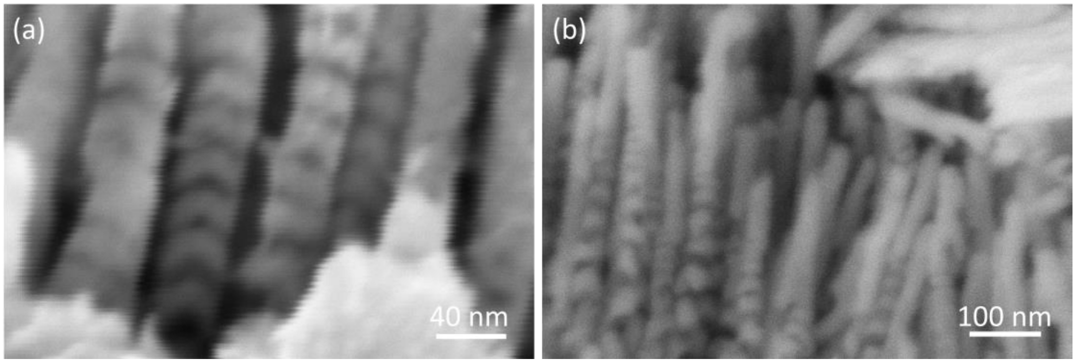

Figure S6: SEM images of two samples with similar growth parameters for the bottom part of the nanowires. (a) and (b) were taken with a stage tilt of 45°.

Because the NiCu layers, especially at the upper end of the nanowires, are thinner than their diameter, that is, they are disk-like, the demagnetizing field along the nanowire is larger than that perpendicular to the nanowire, which makes the easy axis more perpendicular to the nanowire considering the shape of the multilayers. In addition, the dipolar interaction between nanowires has been reported to favor an easy axis perpendicular to the wires [4]. Thus, we chose to apply the magnetic field perpendicular to the nanowire.

4 Detailed description of STT-assisted resistance switching in multilayered nanowires

The asymmetric structure of the magnetic nanowires also contributes to the STT-assisted phenomena when a large current (I) is applied across the long axis of the nanowires. When electrons go through the ferromagnet, they are polarized to the spin direction in parallel with the ferromagnet in a very short distance (typically on the scale of 1 nm [5]) and simultaneously apply a STT on the surface of the ferromagnet to maintain the conservation of the spin angular momentum of the whole system. In our multilayered nanowires, according to current direction in

Figure 3a in the main text, the fixed layer can be deduced to be on the top of the free layers; thus, it is the topmost long NiCu segment. This is consistent with the previous reports on magnetic properties of multilayered nanowires that the long segment of a magnetic layer presents relatively large coercivity as well as net angular momentum and usually preserves its magnetization during the resistance switch process. Besides, the thin NiCu layers are usually considered as the free layers. According to the Landau–Lifshitz–Gilbert–Slonczewski (LLGS) dynamics, the direction of magnetization of thin NiCu layer(s) will be reversed when the net STT torque applied by the incident, transmitted, and reflected electrons has the opposite direction with that of the magnetization of the thin NiCu layer(s) and, at the same time, exceeds a threshold that is set by a competition between STT and Gilbert damping [5].

In our nanowire-based device, when the electrons flow from the thick topmost NiCu layer to the thin NiCu layer, whose magnetization is antiparallel with the thick topmost NiCu layer, the polarized electrons flowing out from the topmost NiCu layer apply a STT to the thin NiCu layer to align its magnetization with the topmost NiCu layer. Under the condition of $I > I_{c+}$, the net STT applied on the thin NiCu layer becomes larger than the angular momentum of the thin NiCu layer. The magnetization of the thin NiCu layer is thus flipped and becomes parallel to that of the thick NiCu layer, leading to the reduction of the resistance. A similar process makes the thin NiCu layer antiparallel to the thick NiCu layer again, when the current is ramped down from above I_{c-} to a negative value. Since the critical current needed to flip the magnetization of thin NiCu layer(s) depends on the total angular momentum of the flipped NiCu layer(s) and the polarized factor of the current, I_{c-} differs from I_{c+} and, therefore, forms the hysteresis in dV/dI.

According to the LLGS equation, the magnitude of the critical current density is proportional to the damping, the total angular momentum of the free magnetic layer and is inversely proportional

to the polarization of the current [5]. The damping term in the LLGS equation increases when the effective magnetic field H_{eff} becomes larger. Here, H_{eff} is determined by the external magnetic field as well as by the exchange stiffness, dipolar field, and anisotropy field caused by the spin–orbit interaction. If we consider a trilayer system with one fixed layer, one free layer and one spacer layer within the “microspin approximation”, the total angular momentum of the free layer and the polarization of the current outgoing from the fixed layer can be considered as unchanged when the effective magnetic field is small. Then, the critical current density increases as H_{eff} becomes larger. Since the spacer layer is thin, the free layer should feel a non-negligible exchange stiffness and the stray field from the fixed layer, whose direction is opposite of the direction of the magnetization of the fixed layer. J. A. Katine et al. have given an estimation of the exchange coupling effect through a 6 nm Cu spacer and calculated that a magnetostatic interaction of roughly 1000 Oe can be felt by the edge of the free layer in their geometry [6]. Therefore, when the trilayer system is in AP configuration and the positive magnetic field is defined to have the same direction as the magnetization of the fixed layer, H_{eff} increases when the external magnetic field becomes more negative.

In our multilayered system, the interaction between NiCu layers and the polarization of the currents becomes much more complex since not only the interaction between the first and the second NiCu layer but also the interactions between various NiCu layers affect H_{eff} on the second NiCu layer. Considering our simplified model in Figure 4, H_{eff} and, thus, I_{c+} monotonously increase as the external magnetic field becomes more negative as long as configuration 1 remains. However, when the third NiCu layer (layer 3 in Figure 4) switches to parallel with the second NiCu layer (configuration 2), the direction of the exchange coupling as well as dipolar stray field exerted by layer 3 to layer 2 is inverted, which decreases H_{eff}. Meanwhile, the direction of the STT exerted

by layer 3 to layer 2 is also changed as shown in Figure 4b, and it further decreases the critical current I_{c+}. Then, H_{eff} increases again as configuration 2 remains. These changes from configuration 1 to 2 can induce one oscillation of I_{c+}.

5 Estimation of the current density in the measured device

With the optimized device fabrication processes, we can characterize the electrical properties of one single nanowire in one device with similar parameters of AAO anodization and nanowire deposition, as shown in Figure S7. According to the *I*–*V* measurement at room temperature (as shown in Figure S7c), the resistance of one NiCu/Cu multilayer nanowire is extracted to be around 704 Ω by the linear fitting. Since the room-temperature resistance of the device shown in our paper is around 130 Ω, we can deduce that the device has contacted about six nanowires that contribute to the current in parallel. Of course, this estimation bears some uncertainties, but it should give the correct order of magnitude. This number is somewhat smaller than the estimation based on the area of the top electrode seen in Figure 1b. For the fabrication of the device shown in Figure 1b we used ion milling to remove the overgrowth and to polish the surface of the AAO template. Therefore, part of the AAO template on the upper surface was removed, and more nanowires reached the polished surface of the AAO template compared to the measured device in Figure 2 Figure 3, whose overgrowth during nanowire deposition was removed by scratching. The surface of the measured device in Figure 2 and Figure 3 is shown in Figure S8. The estimated number of nanowires that reach the surface or are close to it below the electrode of the measured device is around 450. In addition, another argument is that not all pillars contribute to the current because of the corrugation of the surface resulting from the slight length variation, visible in Figure 1b as well as in the SEM image in Figure S8.

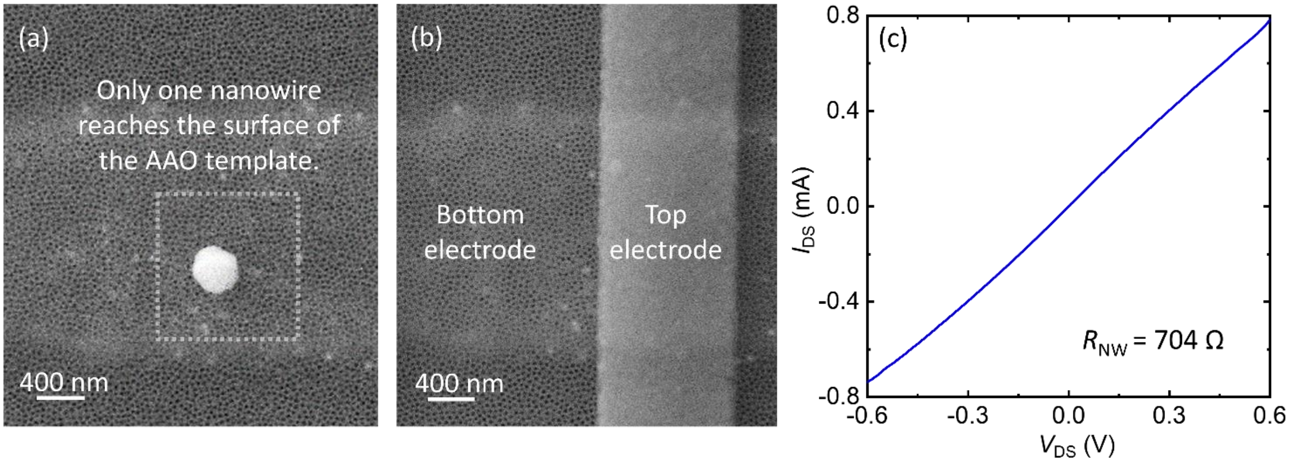

Figure S7: (a) SEM image after deposition of a nanowire array. Only one nanowire is observed to reach the surface of the AAO template and, thus, can be contacted by the top electrode in the further device fabrication processes. (b) SEM image after the fabrication of the top electrode. (c) *I*–*V* curve of one single NiCu/Cu multilayer nanowire at room temperature.

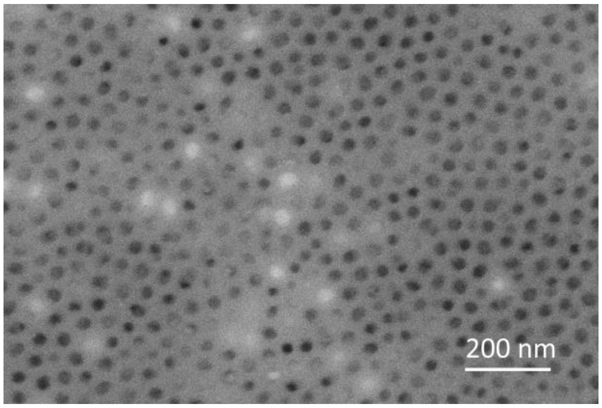

Figure S8: SEM image of the surface of the measured sample in Figure 2 and Figure 3 after nanowire deposition and scratching. 15 nanowires can be observed in the area of 1.33 μm^2.

The diameter of the pores mainly ranges from 30 to 40 nm and is centered at 35 nm. Therefore, the contact area of this device is estimated to be around 5770 nm^2. According to Figure 3 in the main text and Figure S13 below, the critical current of the current-induced spin flip and nonuniform excitation ranges from 1 to 3 mA; thus, the current density is of the order of 10^7 A/cm^2.

6 Raw data before background subtraction

We used four-point measurements in our low-temperature experiments to exclude the resistance contribution of the cabling of the cryostat. The four-point measurement in our experiment was done by bonding twice on each electrode, as shown in Figure S9. In this measurement, the contributions of Ti/Au bottom electrode and Al top electrode and the contact resistances cannot be determined from the measured resistance. The resistivity of Au and Al thin films of that thickness (t_{Au} = 50 nm, t_{Al} = 180 nm) deposited with our methods are very low. From the experience of our lab, the resistances of the Ti/Au and Al electrodes are estimated to be at most 1 Ω, which is much lower than the resistance of the contacted nanowires and, thus, can be neglected in the measurements. The contact resistance of the measured device is also much lower than the device resistance based on the following reasons: (i) The contacts between the nanowires contributing to the current and metal electrodes are ohmic according to the *I*–*V* curve and *I*–d*V*/d*I* curves. (ii) We have fabricated a Ni nanowire-based device by similar processes. A Ni nanowire-based device with a small area of contact (200 nm wide and ca. 1 μm long) and a few contacted nanowires has a device resistance of about 10 Ω. The resistance of Ni nanowires is estimated to be in the range of several ohms; therefore, the total resistance of the electrodes and the contacts is several ohms at maximum.

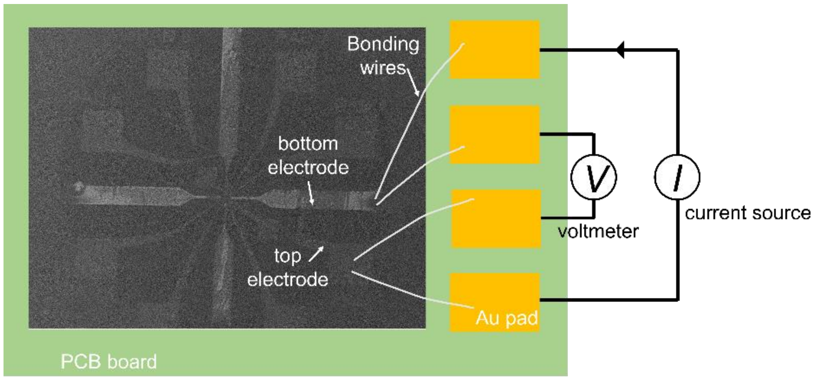

Figure S9: Schematic diagram of the four-point measurements at low temperature.

In our measurements, we used a Yokogawa GS200 DC voltage/current source as the DC supply and a HF2LI lock-in amplifier from Zurich Instruments to measure the differential resistance d*V*/d*I*. The frequency of the lock-in amplifier was set to be 4531 Hz. The raw data of the DC *I*–*V* curve and the 2D color map of d*V*/d*I* are shown in Figure S10a and Figure S10b, respectively. The resistance changes caused by the STT effects are difficult to observe in the *I*–*V* curve because of the large thermal effect on the resistance and small amplitude of the STT-induced resistance change. It is a widely used method to demonstrate small resistance changes by the d*V*/d*I* characteristic as we do in Figure 3. As indicated by the red arrows in Figure S10b, the modulated critical current can also be clearly observed in the raw data but with less contrast compared with the reduced d*V*/d*I* ($(dV/dI)_{red}$) 2D color map in Figure 3b.

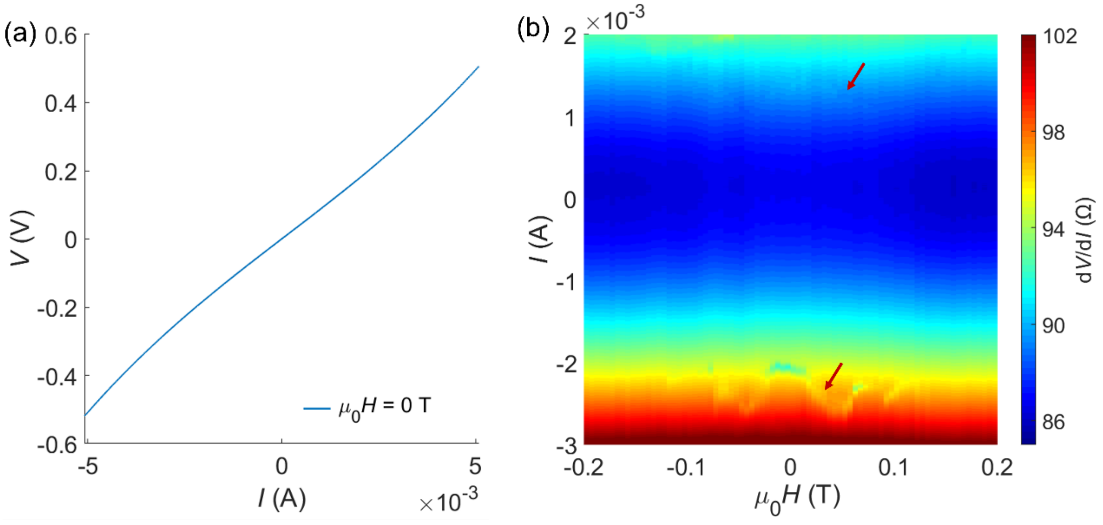

Figure S10: (a) I–V curve under zero magnetic field measured using a Yokogawa GS200 DC voltage/current source. (b) 2D color map of the unnormalized dV/dI of Figure 3b. To better show the features, the modulated critical currents are indicated by the red arrows.

As shown in Figure S11, there is a parabolic background in the dV/dI-vs-I curves, which might result from Joule heating effects caused by the high current density.

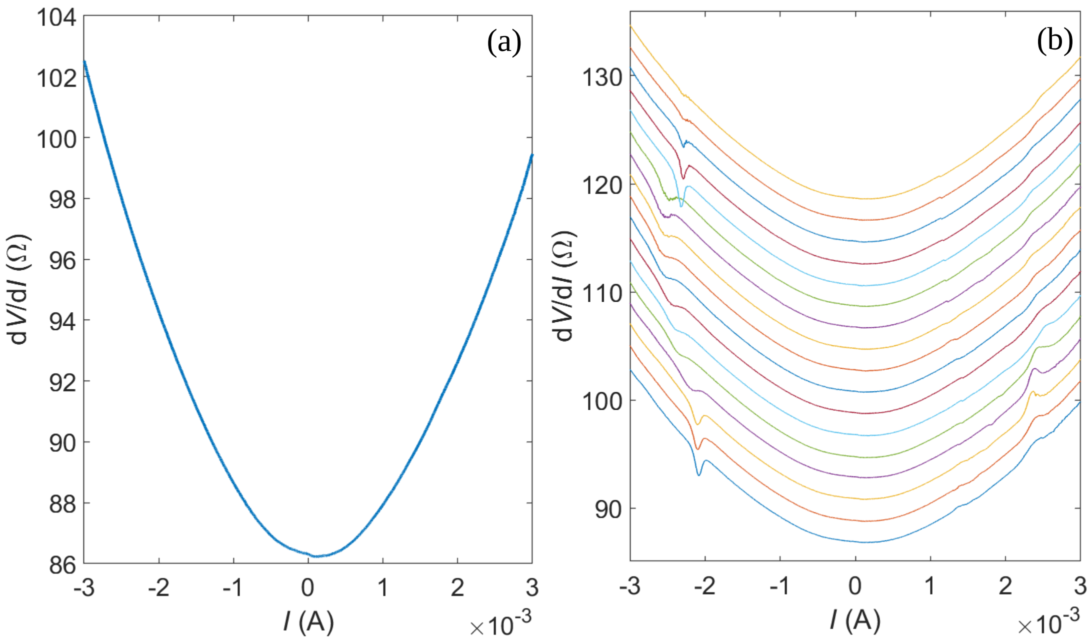

Figure S11: Differential resistance (dV/dI) as a function of the current (I) under different external magnetic fields (μ_0H). The external magnetic field was first set to 2 T and then swept down. (a) is measured under $\mu_0H = -0.3$ T. No current-induced features can be observed. (b) shows a series of curves measured between $\mu_0H = 0$ and $\mu_0H = 80$ mT. To present the current-induced features clearer, an offset of 2 Ω is applied between neighboring curves, and the difference of μ_0H between neighboring curves is 5 mT. The bottommost and topmost curves were measured at $\mu_0H = 0$ mT μ_0H = and 80 mT, respectively. The voltage was ramped down during the measurements.

7 2D color maps of (dV/dI)$_{red}$ over a larger current range and under upsweep of magnetic field

Under larger current, the impact of the other NiCu layers underneath the third NiCu layer might become more pronounced and lead to complex STT effects as observed in the hysteresis of (dV/dI)$_{red}$ and 2D color maps in Figure S12. Since this work does not aim to explain all the complex phenomena, we leave the detailed understanding as an open question for further study.

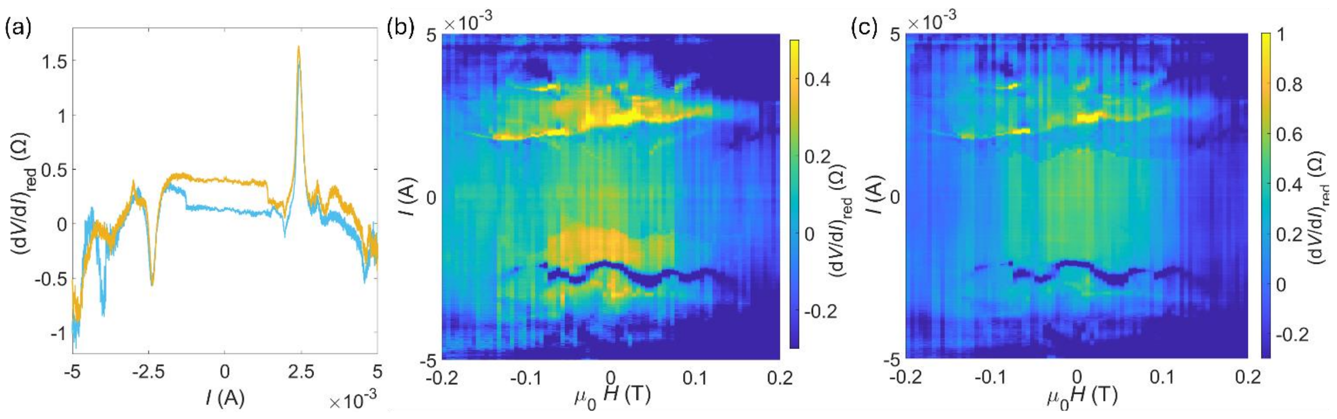

Figure S12: (a) The hysteresis of the reduced differential resistance $(\mathrm{d}V/\mathrm{d}I)_{\mathrm{red}}$ and (b, c)the 2D color maps of $(\mathrm{d}V/\mathrm{d}I)_{\mathrm{red}}$ as a function of magnetic field and current under larger current sweep compared with Figure 3. The current was ramped up in (b) and was ramped down in (c).

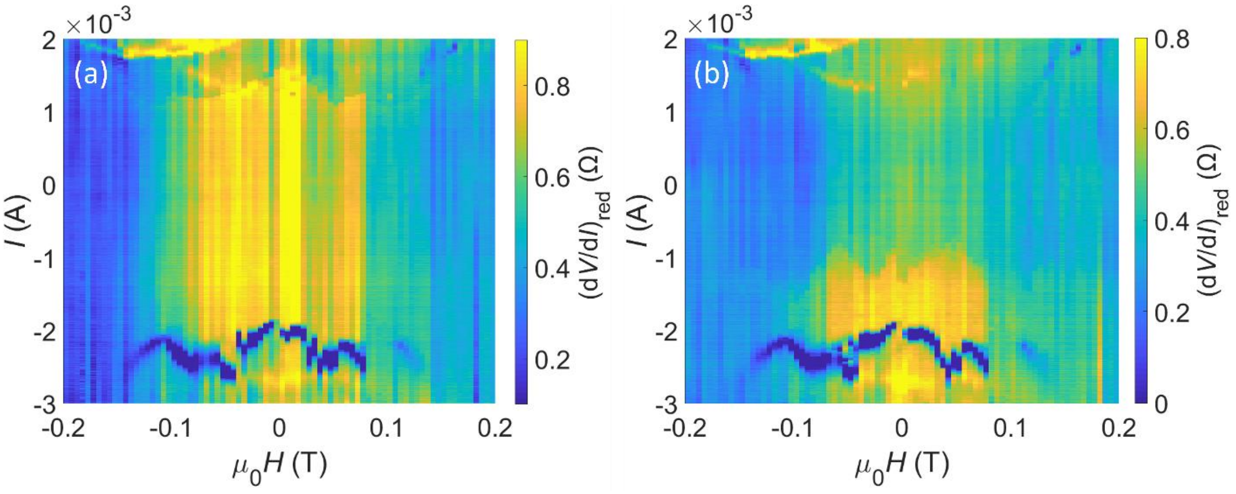

Figure S13: 2D color map of $(\mathrm{d}V/\mathrm{d}I)_{\mathrm{red}}$ under upsweep of magnetic field. (a) was measured when the current I was swept up and (b) was measured during sweeping down.

8 Potential influence of the dipolar interactions between nanowires

The thickness of AlO_x between the neighboring nanowires is at least 15 nm, which makes the exchange coupling weak, and dipolar stray fields play the main role in the interaction between the

nanowires. The dipolar interaction between nanowires has been reported to favor an easy axis of the magnetic nanowire array perpendicular to the wires [4].

However, although the dipolar interactions between the nanowires are non-negligible for the collective magnetic properties of the nanowire array, considering the size of the total device, the dipolar stray field produced by one thin NiCu is negligible because of its small volume and, thus, small saturation magnetization M_s. Therefore, the unsynchronized flip of the free layer in different nanowires cannot produce pronounced effects on the critical current. In contrast, the exchange coupling between the neighboring NiCu layers in one nanowire is strong according to the AP configuration under zero magnetic field.

9 VSM measurements for the electrodeposited nanowire arrays in an AAO template

The magnetic properties of the multilayered nanowires can be controlled by varying the deposition parameters. Through systematical VSM characterization, we measured the hysteresis curve and the Curie temperature of nanowire arrays deposited with various concentrations of Cu in the electrolyte (the concentration of Ni was kept constant), from which we obtained NiCu/Cu multilayered nanowires having different proportions of Ni. The samples studied in the VSM measurements below were cut to 3 × 3 mm^2 size to fit the sample holder, and the nanowire array covered the whole surface of the Si substrate to increase the signal-to-noise ratio of the measurements. Before the VSM measurement, the AAO template was removed by etching in NaOH solution. As shown in Figure S14, the Curie temperature increases as the proportion of Ni in the nanowires becomes larger.

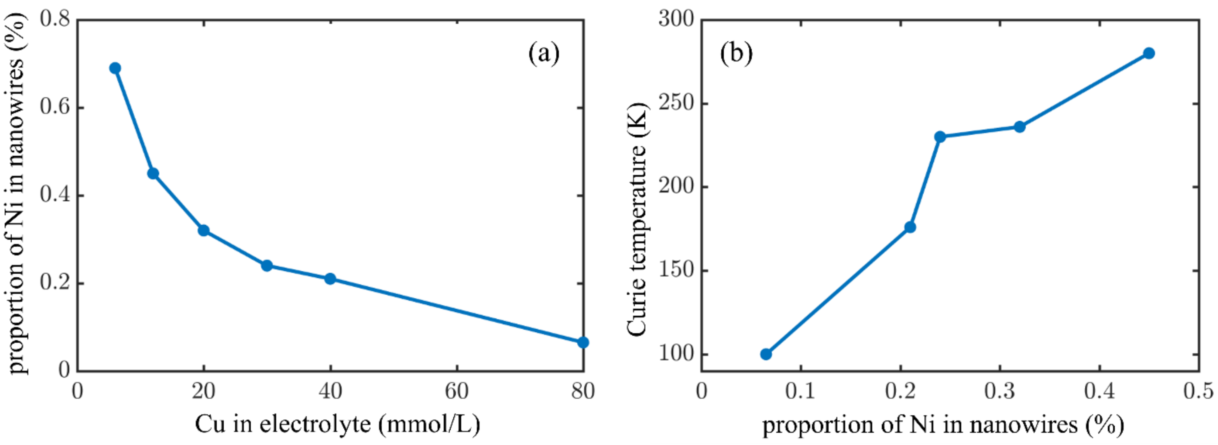

Figure S14: (a) The relationship between the proportion of Ni in the nanowires and the concentration of Cu in the electrolyte of electrodeposition. The proportion of Ni was measured by EDS in the SEM. (b) The dependence of the Curie temperature on the proportion of Ni in the nanowires.

Figure S15 shows the VSM measurements for a nanowire array with similar parameters of AAO template fabrication and nanowire deposition. We note that the magnetic moment of the nanowire array does not show multiple steps in the magnetic field sweep, but instead a gradual change in the slope of the curve. Therefore, the slight wire-to-wire variation among the large number of the nanowires as well as the thickness change of the NiCu layers along the nanowires cannot be characterized through VSM measurements, which only reflects the collective properties of the nanowire array. Characterization of the magnetic properties of individual or a few nanowires is not achievable through VSM measurements in our equipment because of the small volume of the nanowires and the finite sensitivity and resolution of the VSM.

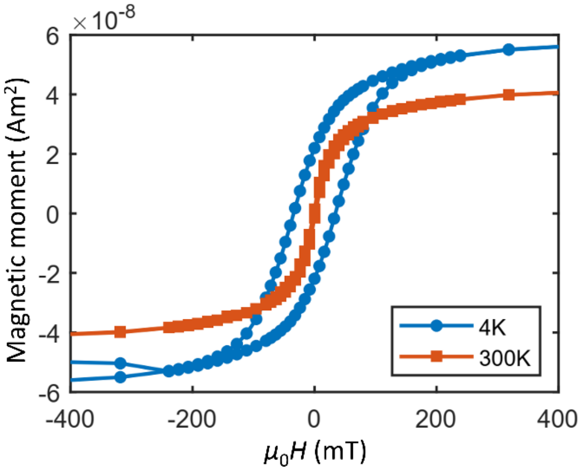

Figure S15: VSM measurements of the nanowire array with similar parameters of AAO template fabrication and nanowire deposition. The pulse cycles were repeated 40 times during nanowire deposition to make sure no bulk overgrowth was on the surface of AAO template. The magnetic field was applied perpendicular to the surface of Si substrate.

Furthermore, the VSM measurements on the NiCu/Cu multilayered nanowire arrays further confirmed that the easy axis of the nanowire is preferred to be perpendicular to the nanowires instead of along the nanowires, as shown in Figure S16. Though the VSM measurements in Figure S16 show that the nanowire array retains weak ferromagnetic properties at room temperature, they cannot ensure that all NiCu are ferromagnetic since the VSM captures only the total magnetic moment of the nanowire array. In addition, the current applied to the nanowires for the electrical measurements increases their temperature. In order to observe the STT effects, it is necessary that all free and fixed layers stay in the ferromagnetic state. Therefore, the electrical measurements were only carried out at 4 K.

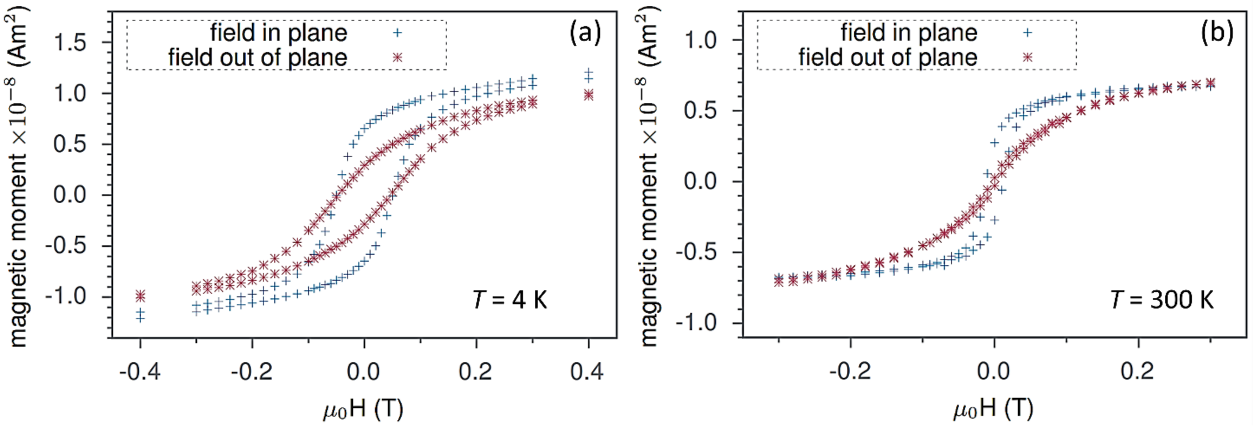

Figure S16: VSM measurements for one nanowire array sample under two directions of magnetic field at (a) 4 K and (b) 300 K. Here, the in-plane direction is parallel to the plane of wafer and, thus, perpendicular to the nanowires. The lower part of the nanowire array was deposited in electrolyte with 20 mmol/L Cu for 15 cycles, and the upper part was fabricated in electrolyte with 40 mmol/L Cu for 15 cycles. The Ni concentration for both electrolytes was kept at 0.2 M, which is the same as for the measured device in Figure 2 and Figure 3.

10 Discussion on the wire-to-wire variations

The wire-to-wire variations are unavoidable during the deposition of nanowire arrays. Both the strong variations from wire to wire in the array or strong thickness variations of the individual NiCu and Cu layers within one nanowire may lead to multiple peaks in the *R*–*H* measurement in Figure 2b. In what follows, we summarize our arguments why the wire-to-wire variation may not cause the multiple steps in the current and the field modulation of the critical current.

(i) We did not see a large difference between the deposited nanowires in our experiments based on the SEM images (Figure 2a and the SEM image of another sample in Figure S17). Since the small number of nanowires contacted here are in a small area, we assume that they are very similar regarding the thickness of the individual layers.

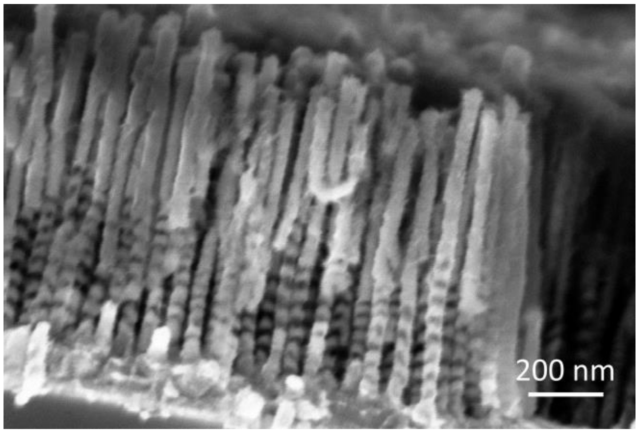

Figure S17: SEM image of another nanowire array sample. The nanowires deposited in this sample underwent ten cycles of NiCu–Cu layer deposition with the same deposition parameters as the measured sample in Figure 2 and Figure 3 at the beginning. After the cycles of NiCu–Cu layer deposition, a long Co segment was deposited by changing the electrolyte and controlling the deposition potential. It shows that the multilayered structure can be efficiently altered and controlled by the deposition parameters.

(ii) The wire-to-wire variations are also unlikely to cause multiple steps in the *R*–*H* measurements because of the synchronizing effect of the dipolar interaction between the nanowires. We note that in the previous works on AAO template-assisted electrodeposition of multilayered nanowires [7,8], the *R*–*H* measurement in most cases did not show multiple steps if the magnetic layers had similar thickness and similar spacer thickness, although differences should exist among the nanowires also in these works. One possible reason for this is that the switching of the magnetization of the nanowires could be synchronized because of the strong dipolar interaction between them, similar to the synchronization behavior of STT nano-oscillators based on nanowire arrays [9,10].

(iii) In principle, both the wire-to-wire variation and the thickness variation along each nanowire might lead to multiple steps in the *R*–*H* measurements if these variations are pronounced to overcome the synchronization effects. However, combined with the results shown in Figure 3, we propose that the difference of the nanowires would not be the main reason for the large modulation

of the critical current. The thickness of AlO_x between the neighboring nanowires is at least 15 nm, which makes the exchange coupling weak, and dipolar stray fields play the main role in the interaction between the nanowires. Although the dipolar interactions between nanowires are not negligible for the collective magnetic properties of the nanowire array, the dipolar stray field produced by one thin NiCu segment is negligible because of its small volume and, thus, small saturation magnetization M_s. Therefore, the unsynchronized flip of one free layer in one nanowire cannot produce the pronounced modulation effect of the critical currents in neighboring nanowires. In contrast, the exchange coupling between the neighboring NiCu layers within one nanowire is strong according to the AP configuration under zero magnetic field and, thus, the modulated critical current as shown in Figure 3 can be obtained.

(iv) In addition, the hysteresis of $(\mathrm{d}V/\mathrm{d}I)_{red}$ as a function of I and the 2D color maps of $(\mathrm{d}V/\mathrm{d}I)_{red}$ (Figure 3) further support that the inhomogeneity of the nanowires is not the most probable reason for the multiple steps in R–H measurements and the modulation of the critical currents in Figure 3. If the multiple steps in the R–H measurements originated from the different switching fields of the contacted nanowires, we would expect to observe multiple abrupt resistance changes in the individual $(\mathrm{d}V/\mathrm{d}I)_{red}$ curves (Figure 3a) and multiple positive critical current (I_{c+}) lines in the 2D color maps of $(\mathrm{d}V/\mathrm{d}I)_{red}$ (Figure 3b) (similar to the unsynchronized case in [9,10]), because each nanowire switches its direction of the magnetization separately. Besides, the changes of the critical currents would be not continuous but more discrete in the color maps in this case. However, the modulation is continuous in the 2D color maps of $(\mathrm{d}V/\mathrm{d}I)_{red}$ (Figure 3).

Therefore, we propose that the multiple peaks in R–H measurements as well as the modulation on the critical currents of the magnetic field in the 2D color maps are more likely caused by the nonuniform and asymmetric structure along the axis of the nanowires.

References

1. Detor, A.; Schuh, C. *Acta Mater.* **2007,** *55,* 371. doi:10.1016/j.actamat.2006.08.032
2. Bograchev, D. A.; Volgin, V. M.; Davydov, A. D. *Electrochim. Acta* **2016,** *207,* 247. doi:10.1016/j.electacta.2016.04.119
3. Roy, S.; Matlosz, M.; Landolt, D. *J. Electrochem. Soc.* **1994,** *141,* 1509–1517. doi:10.1149/1.2054954
4. Encinas-Oropesa, A.; Demand, M.; Piraux, L.; Huynen, I.; Ebels, U. *Phys. Rev. B* **2001,** *63,* 104415. doi:10.1103/PhysRevB.63.104415
5. Brataas, A.; Kent, A. D.; Ohno, H. *Nat. Mater.* **2012,** *11,* 372–381. doi:10.1038/nmat3311
6. Katine, J.; Albert, F.; Buhrman, R.; Myers, E.; Ralph, D. *Phys. Rev. Lett.* **2000,** *84,* 3149. doi:10.1103/PhysRevLett.84.3149
7. Huang, X.; Tan, L.; Cho, H.; Stadler, B. J. H. *J. Appl. Phys.* **2009,** *105,* 07d128. doi:10.1063/1.3075990
8. Fedorov, F. S.; Mönch, I.; Mickel, C.; Tschulik, K.; Zhao, B.; Uhlemann, M.; Gebert, A.; Eckert, J. *J. Electrochem. Soc.* **2012,** *160,* D13–D19. doi:10.1149/2.006302jes
9. Kaka, S.; Pufall, M. R.; Rippard, W. H.; Silva, T. J.; Russek, S. E.; Katine, J. A. *Nature* **2005,** *437,* 389–392. doi:10.1038/nature04035
10. Romera, M.; Talatchian, P.; Tsunegi, S.; Abreu Araujo, F.; Cros, V.; Bortolotti, P.; Trastoy, J.; Yakushiji, K.; Fukushima, A.; Kubota, H. *Nature* **2018,** *563,* 230–234. doi:10.1038/s41586-018-0632-y
